# Supplementary figures and images for: Drosophila models uncover substrate channeling effects on phospholipids and sphingolipids in peroxisomal biogenesis disorders
Source: PLoS One. 2025 Jun 11;20(6):e0324143. doi: 10.1371/journal.pone.0324143 (PMC12157166; doi:10.1371/journal.pone.0324143)

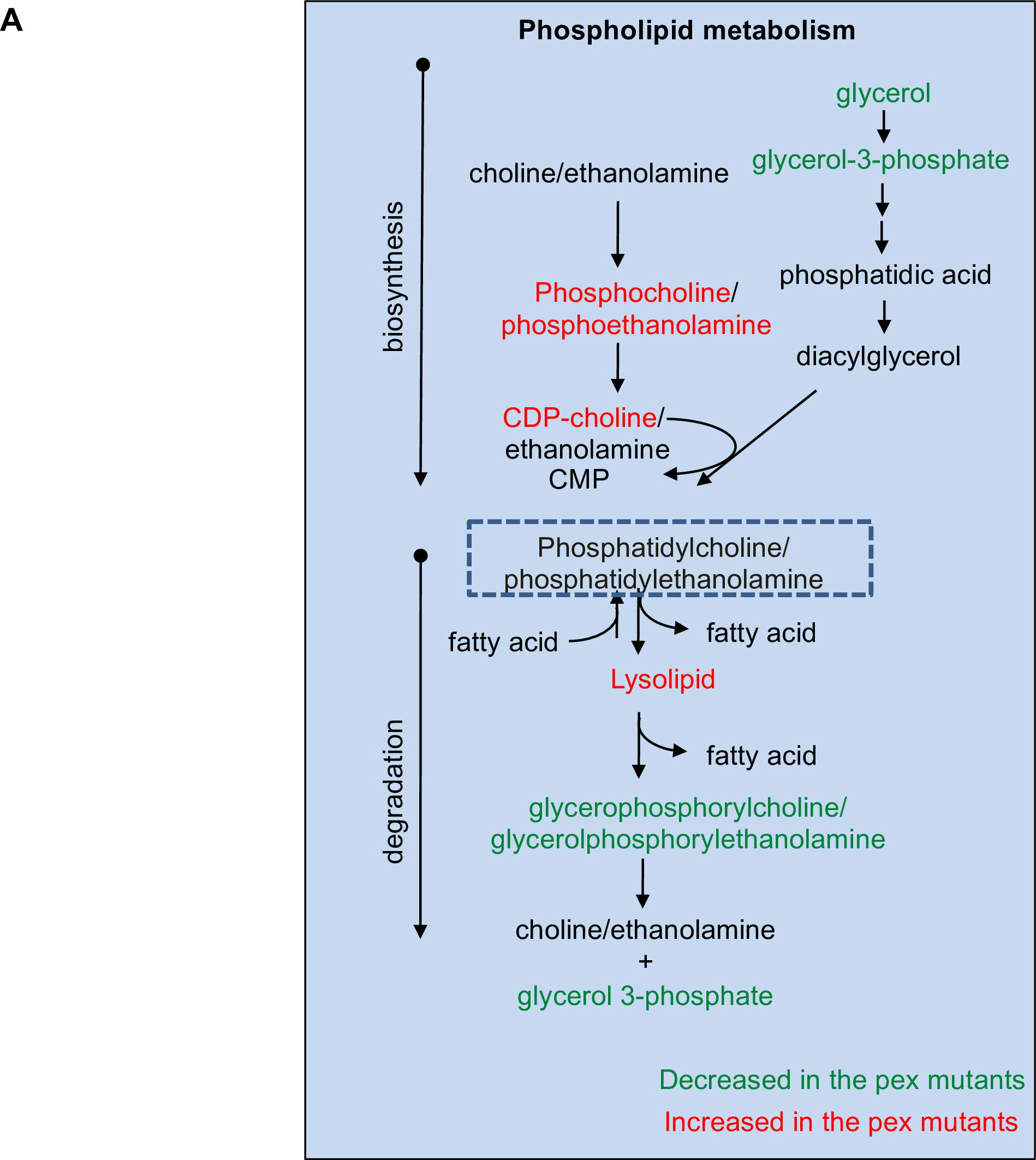

Supplement: Fig S1 — In our previous metabolomic analysis of pex2 and pex16 mutants phospholipid abnormalities were identified which included a decrease in glycerol and glycerol-3-phosphate. Diacylglycerol is converted to phosphatidlycholine (with the addition of choline) or phosphatidylethanolamine (with the addition of ethanolamine) and the choline and ethanolamine precursors appear to be increased in the pex mutants. Also noted was an apparent excess of lysolipids and reduces levels of phospholipid breakdown products glycerophophorylcholine/glycerolethanolamine and glycerol 3-phosphate. Of note the levels of phosphatidylcholine and phosphatidylethanolamine themselves were normal, though the specific chain lengths were not analyzed in these previous studies. (TIF) [file pone.0324143.s001.tif]

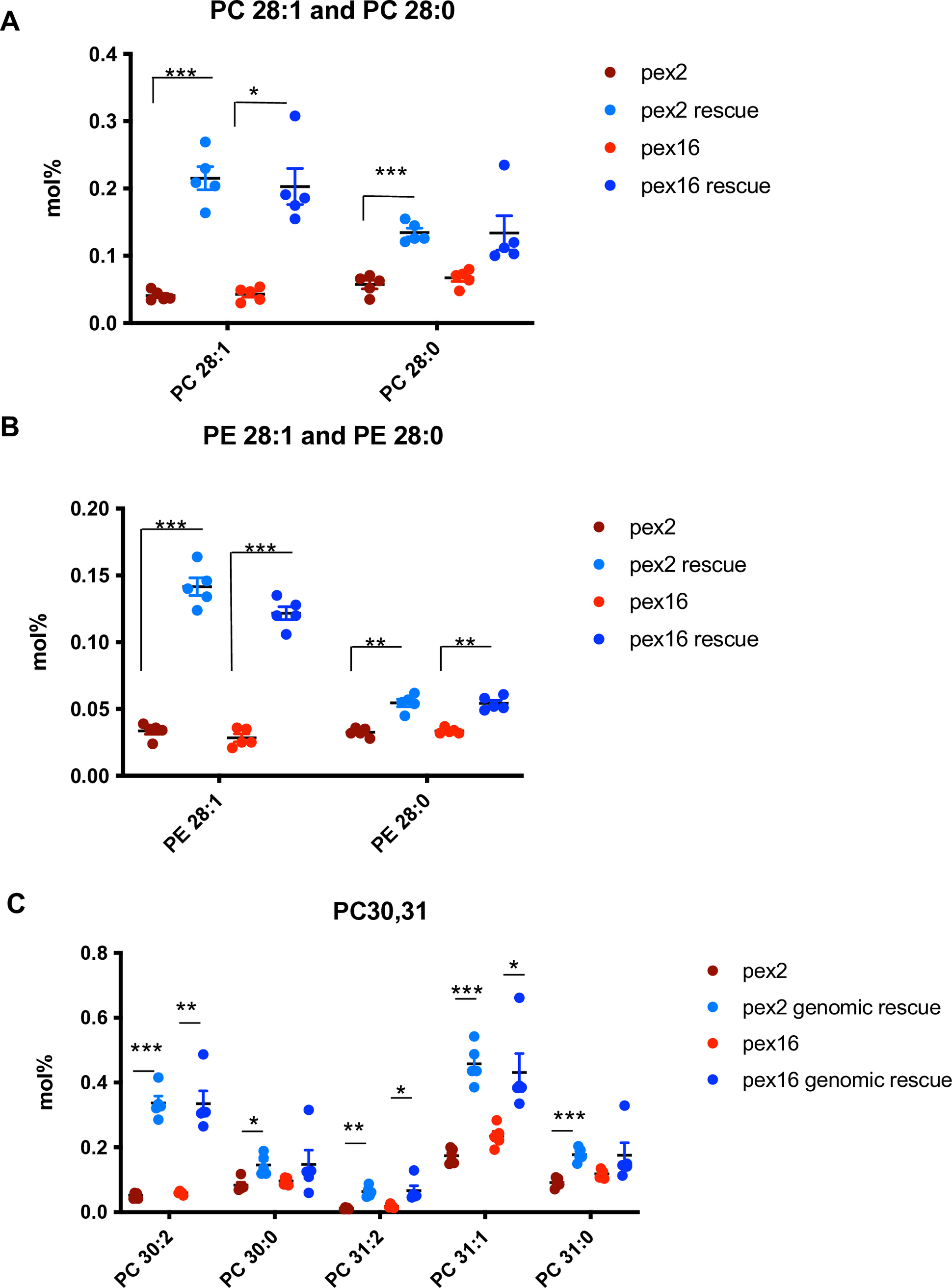

Supplement: Fig S2 — A. Total levels in mol% of PC 28:1 and PC 28:0 Phosphatidylcholine (PC) in pex2 and pex16 larvae shows dramatic and significant decreases in intermediate chain phospholipids. For PC 28:1, pex2 mutant larvae compared to pex2 rescue (ratio pex2/pex2 rescue: ratio 0.1889,p = 0.0006), and a dramatic decrease in pex16 mutant larvae compared to pex16 rescue (pex16/pex16 rescue: ratio 0.211, p = 0.003). For PC 28:0, pex2 mutant larvae compared to pex2 rescue (ratio pex2/pex2 rescue: ratio 0.427,p = 0.0002), and a non-significant decrease in pex16 mutant larvae compared to pex16 rescue (pex16/pex16 rescue: ratio 0.502, p = 0.0555). B. Total levels in mol% of PE 28:1 and PE 28:0 Phosphatidylethanolamine (PE) in pex2 and pex16 larvae shows dramatic and significant decreases in intermediate chain phospholipids. For PE 28:1, pex2 mutant larvae compared to pex2 rescue (ratio pex2/pex2 rescue: ratio 0.2369,p=0.0003), and a dramatic decrease in pex16 mutant larvae compared to pex16 rescue (pex16/pex16 rescue: ratio 0.233, p=7.97E-05). For PE 28:0, pex2 mutant larvae compared to pex2 rescue (ratio pex2/pex2 rescue: ratio 0.594,p=0.0027), and a non-significant decrease in pex16 mutant larvae compared to pex16 rescue (pex16/pex16 rescue: ratio 0.624, p=0.0022). C. Total levels in mol% of PC 30:2, PC 30:0, PC 31:2, PC31:1, and PC 31:0 Phosphatidylcholine (PC) in pex2 and pex16 larvae showing dramatic and significant decreases in intermediate chain phospholipids. For PC 30:2, pex2 mutant larvae compared to pex2 rescue (ratio pex2/pex2 rescue: ratio 0.1545,p = 0.0002), and a dramatic decrease in pex16 mutant larvae compared to pex16 rescue (pex16/pex16 rescue: ratio 0.1775, p = 0.0018). For PC 30:0, pex2 mutant larvae compared to pex2 rescue (ratio pex2/pex2 rescue: ratio 0.5748,p = 0.0303), and a dramatic decrease in pex16 mutant larvae compared to pex16 rescue (pex16/pex16 rescue: ratio 0.6542, p = 0.3048). For PC 31:2, pex2 mutant larvae compared to pex2 rescue (ratio pex2/pex2 rescu [file pone.0324143.s002.tif]

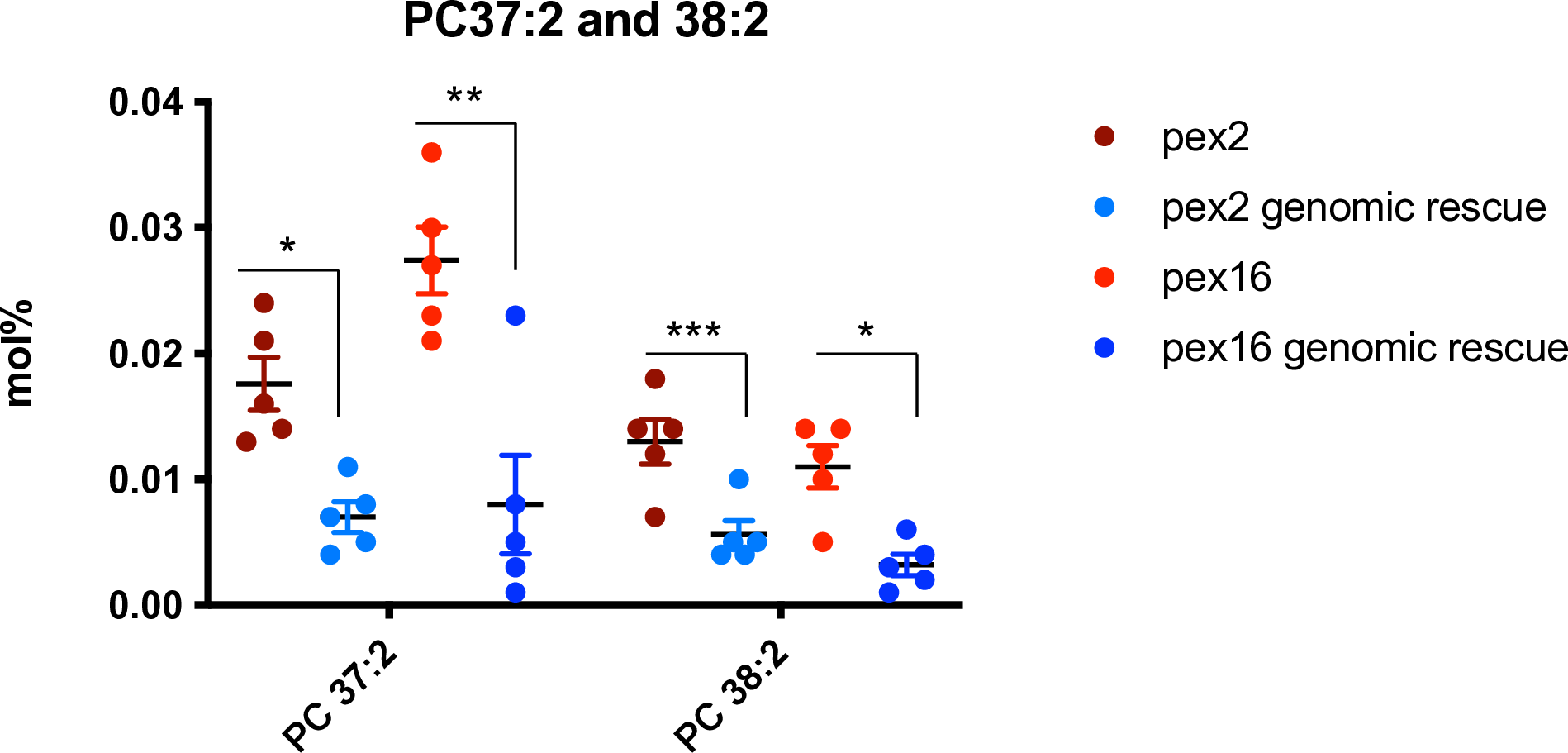

Supplement: Fig S3 — Total levels in mol% of PC 37:2: and PC 38:2 Phosphatidylcholine (PC) in pex2 and pex16 larvae shows dramatic and significant increases in long chain phospholipids. For PC 37:2, pex2 mutant larvae compared to pex2 rescue (ratio pex2/pex2 rescue: ratio 2.473,p = 0.0183), and a dramatic increase in pex16 mutant larvae compared to pex16 rescue (pex16/pex16 rescue: ratio 3.446, p = 0.002). For PC 38:2, pex2 mutant larvae compared to pex2 rescue (ratio pex2/pex2 rescue: ratio 2.303,p = 0.0426), and a non-significant decrease in pex16 mutant larvae compared to pex16 rescue (pex16/pex16 rescue: ratio 3.243, p = 0.0193). (TIF) [file pone.0324143.s003.tif]

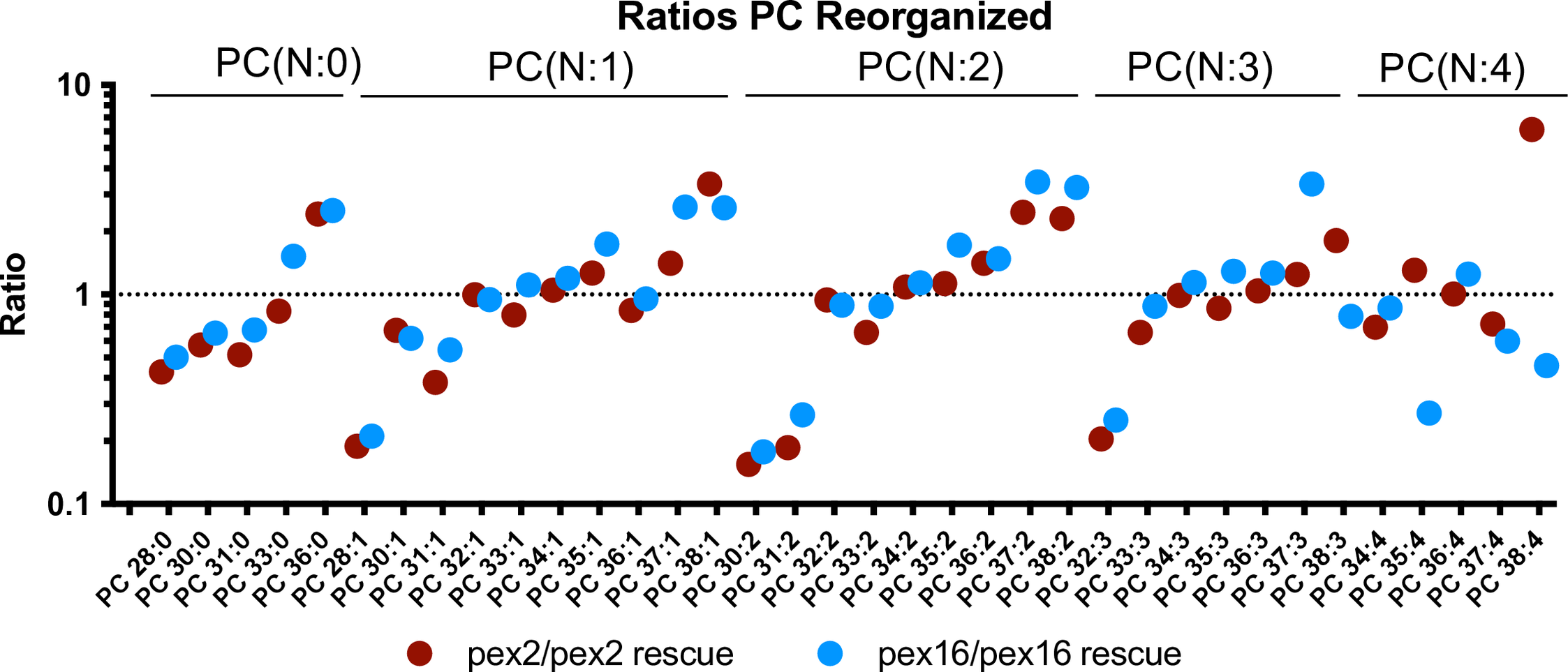

Supplement: Fig S4 — The same ratios depicted in Fig 3A is reorganized based on the total number of unsaturations, organized from (N:0) up to (N:4) showing that across each class the ratios or pex2/pex2 rescue and pex16/pex16 rescue increases as chain-length increases. Clear trends are not observed for the number of unsaturations themselves. (TIF) [file pone.0324143.s004.tif]

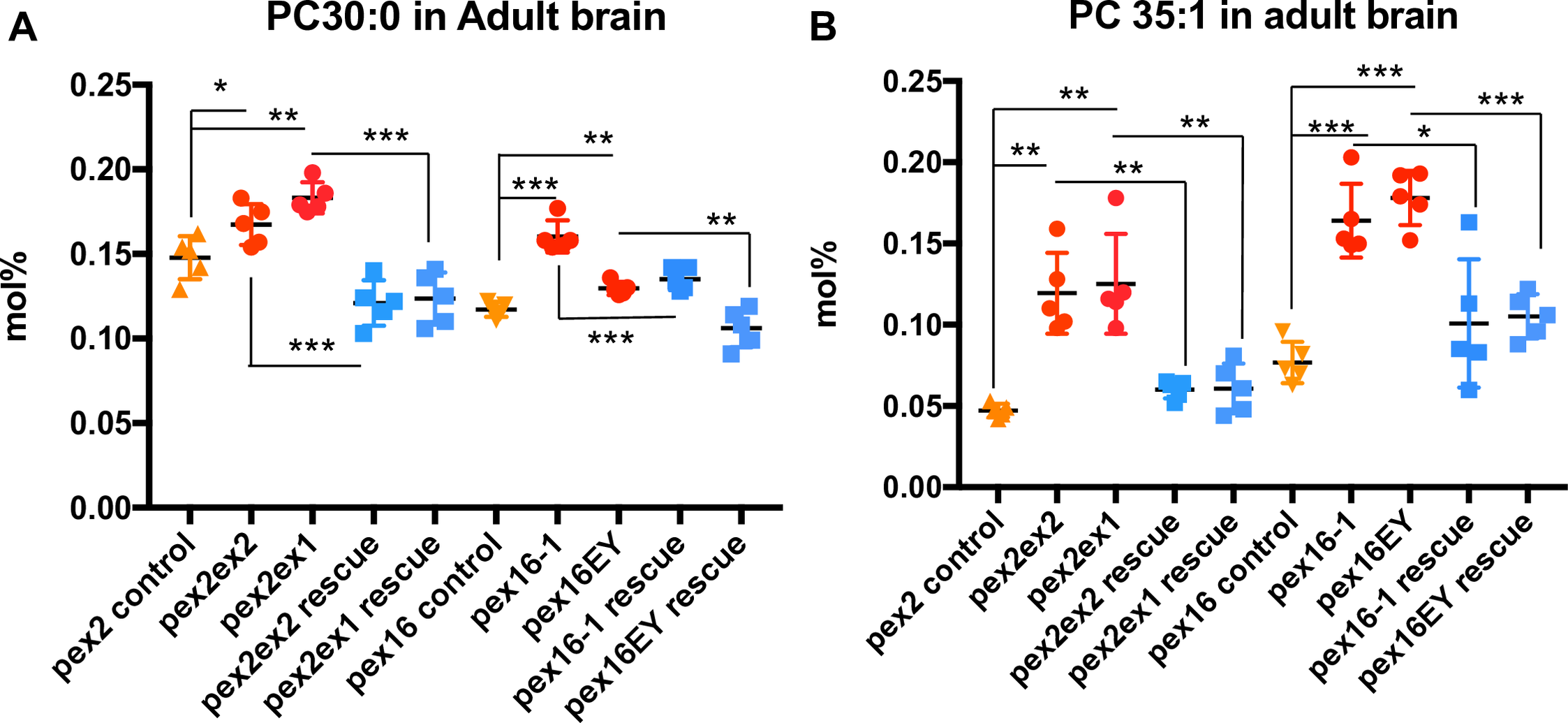

Supplement: Fig S5 — A. Levels in mol% of PC 30:0 in pex2 and pex16 mutant brains show significant increases in pex mutant brains. For PC 30:0 in pex22 brain and pex21 brain, levels are increased compared to controls (ratio 1.131, 1.238 respectively, p = 0.038, p = 0.001 respectively) and pex22 brain compared to rescue (ratio 1.383, p < 0.001), and pex21 compared to rescue (ratio 1.483, p < 0.001). For PC 30:0 in pex161 and pex16EY brain, levels are increased compared to controls (ratio 1.368, 1.107 respectively, p < 0.001, p = 0.001 respectively). And pex161 brain compared to rescue (ratio 1.186, p = 0.002), and increased pex16EY compared to rescue (ratio 1.222, p = 0.008). B. Levels in mol% of PC 35:1 in pex2 and pex16 mutant brains shows significant increases in pex mutant brains. For PC 35:1 in pex22 brain and pex21 brain, levels are increased compared to controls (ratio 2.529, 1.653 respectively, p = 0.003, p = 0.004 respectively) and pex22 brain compared to rescue (ratio 1.985, p = 0.005) as well as pex21 compared to rescue (ratio 2.059, p = 0.006). For PC 35:1 in pex161 and pex16EY brain, levels are increased compared to controls (ratio 2.129, 2.310 respectively, p < 0.001, p < 0.001 respectively). And pex161 brain compared to rescue (ratio 1.629, p = 0.019), and for pex16EY compared to rescue (ratio 1.690, p < 0.001). (TIF) [file pone.0324143.s005.tif]

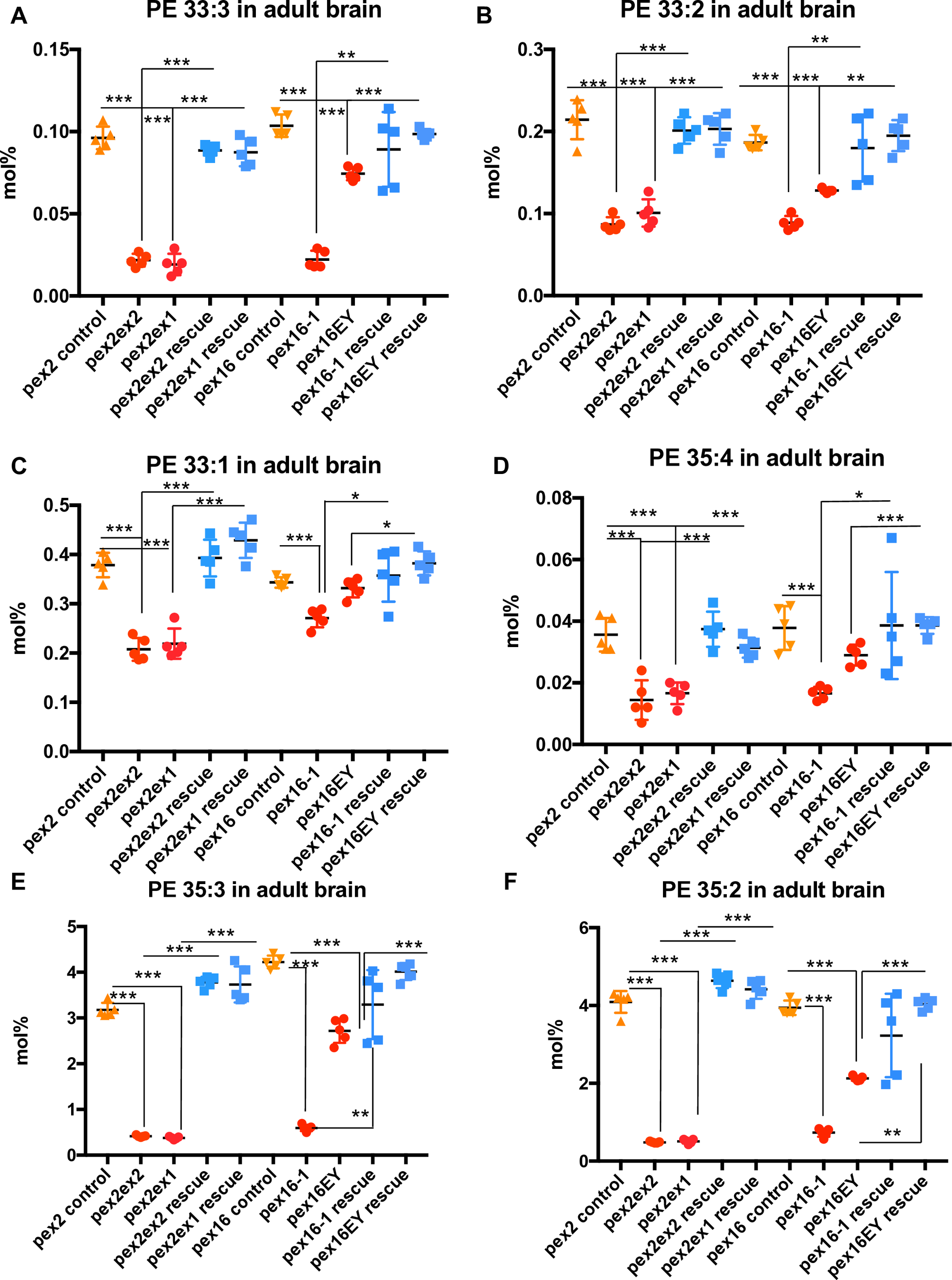

Supplement: Fig S6 — A. Levels in mol% of PE 33:3 in pex2 and pex16 mutant brains show significant decreases in pex mutant brains. For PE 33:3 in pex22 brain and pex21 brain, levels are dramatically reduced compared to controls (ratio 0.225, 0.200 respectively, p < 0.001, p < 0.001 respectively) and pex22 brain compared to rescue (ratio 0.244, p < 0.001), and pex21 compared to rescue (ratio 0.220, p < 0.001). For PE 33:3 in pex161 and pex16EY brain, levels are decreased compared to controls (ratio 0.214, 0.718 respectively, p < 0.001, p < 0.001 respectively). And pex161 brain compared to rescue (ratio 0.249, p = 0.002), and for pex16EY compared to rescue (ratio 0.756, p < 0.001). B. Levels in mol% of PE 33:2 in pex2 and pex16 mutant brains show significant decreases in pex mutant brains. For PE 33:2 in pex22 brain and pex21 brain, levels are dramatically reduced compared to controls (ratio 0.403, 0.469 respectively, p<0.001, p<0.001 respectively) and pex22 brain compared to rescue (ratio 0.430, p<0.001), and pex21 compared to rescue (ratio 0.496, p<0.001). For PE 33:2 in pex161 and pex16EY brain, levels are decreased compared to controls (ratio 0.475, 0.688 respectively, p<0.001, p<0.001 respectively). And pex161 brain compared to rescue (ratio 0.494, p=0.006), and for pex16EY compared to rescue (ratio 0.658, p=0.001). C. Levels in mol% of PE 33:1 in pex2 and pex16 mutant brains show significant decreases in pex mutant brains. For PE 33:3 in pex22 brain and pex21 brain, levels are dramatically reduced compared to controls (ratio 0.549, 0.578 respectively, p<0.001, p<0.001 respectively) and pex22 brain compared to rescue (ratio 0.528, p<0.001), and pex21 compared to rescue (ratio 0.510, p<0.001). For PE 33:3 in pex161 levels are decreased compared to controls (ratio 0.789, p<0.001). And pex161 brain compared to rescue (ratio 0.758, p=0.019). For the pex16EY the difference was not significant from control, but was reduced compared to rescue (ratio 0.868, p=0.006). D. Levels in mol% of PE [file pone.0324143.s006.tif]

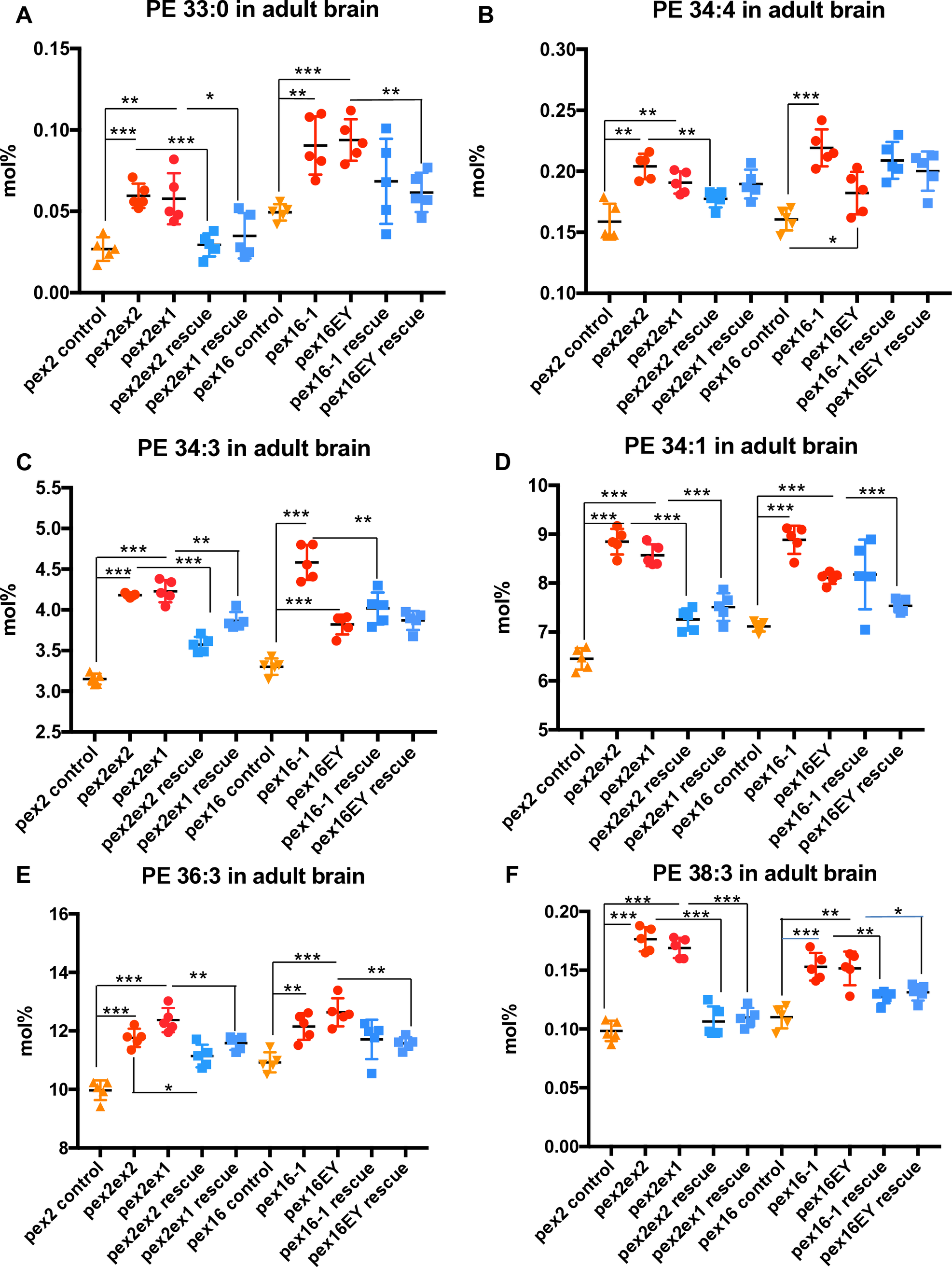

Supplement: Fig S7 — A. Levels in mol% of PE 33:0 in pex2 and pex16 mutant brains show significant increases in pex mutant brains. For PE 33:0 in pex22 brain and pex21 brain, levels are increased compared to controls (ratio 2.227, 2.153 respectively, p < 0.001, p = 0.009 respectively) and pex22 brain compared to rescue (ratio 2.032, p < 0.001), and pex21 compared to rescue (ratio 1.644, p = 0.044). For PE 33:0 in pex161 and pex16EY brain, levels are increased compared to controls (ratio 1.834, 1.900 respectively, p = 0.005, p = 0.001 respectively) and for pex16EY compared to rescue (ratio 1.518, p = 0.003). B. Levels in mol% of PE 34:4 in pex2 and pex16 mutant brains show significant decreases in pex mutant brains. For PE 34:4 in pex22 brain and pex21 brain, levels are increased compared to controls (ratio 1.285, 1.202 respectively, p=0.001, p=0.005 respectively) and pex22 brain compared to rescue (ratio 1.151, p=0.002). For PE 34:4 in pex161 and pex16EY brain, levels are increased compared to controls (ratio 1.366, 1.136 respectively, p<0.001, p=0.048 respectively) but no differences from rescue. C. Levels in mol% of PE 34:3 in pex2 and pex16 mutant brains show significant increases in pex mutant brains. For PE 34:3 in pex22 brain and pex21 brain, levels are increased compared to controls (ratio 1.327, 1.343 respectively, p<0.001, p<0.001 respectively) and pex22 brain compared to rescue (ratio 1.170, p<0.001), and pex21 compared to rescue (ratio 1.094, p=0.002). For PE 34:3 in pex161 and pex16EY brain, levels are increased compared to controls (ratio 1.388, 1.157 respectively, p<0.001, p<0.001 respectively) and for pex161 compared to rescue (ratio 1.141, p=0.003). D. Levels in mol% of PE 34:1 in pex2 and pex16 mutant brains show significant increases in pex mutant brains. For PE 34:1 in pex22 brain and pex21 brain, levels are increased compared to controls (ratio 1.371, 1.328 respectively, p<0.001, p<0.001 respectively) and pex22 brain compared to rescue (ratio 1.219, p<0.001), and pex [file pone.0324143.s007.tif]

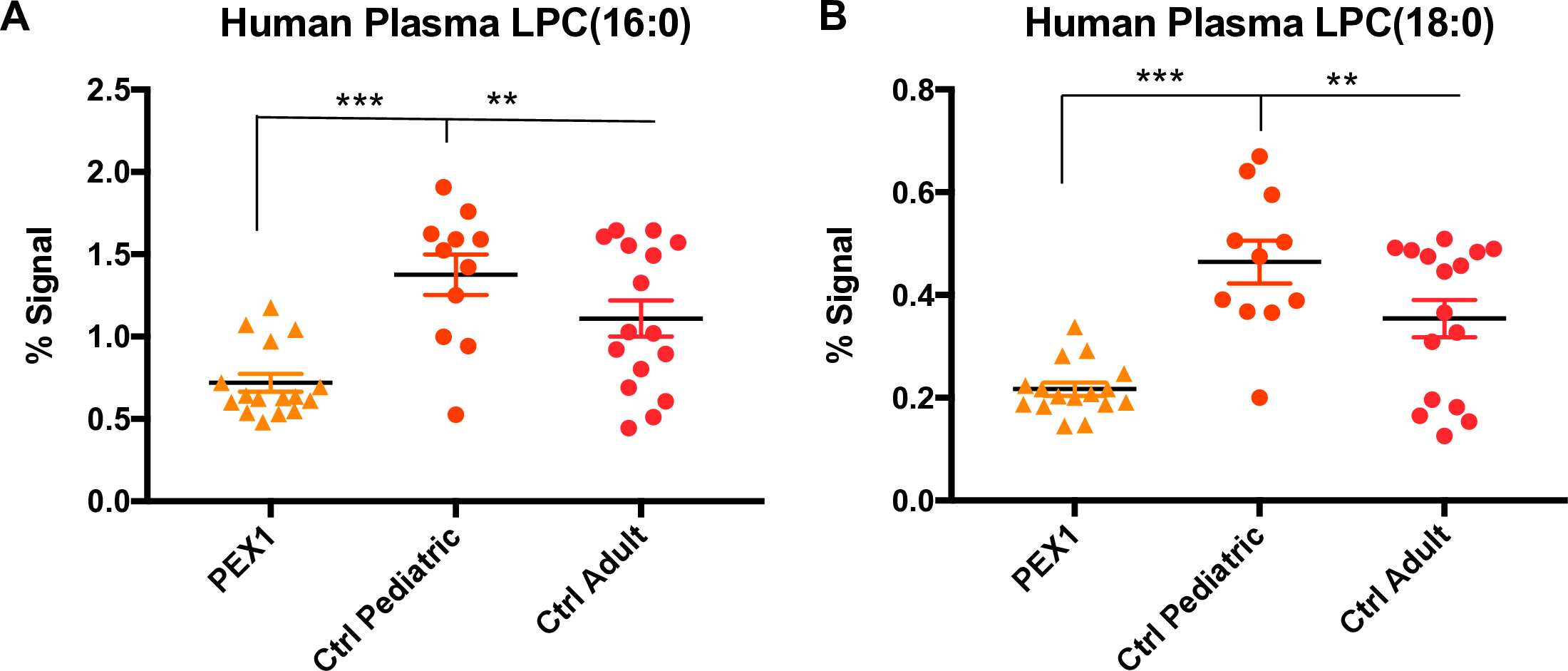

Supplement: Fig S8 — A. Human Plasma LPC(16:0) is decreased in patients with PEX1 mutations compared to pediatric (ratio 0.523, p < 0.001) and adult controls (ratio 0.656, p < 0.001). B. Human Plasma LPC(18:0) is decreased in patients with PEX1 mutations compared to pediatric (ratio 0.467, p < 0.001) and adult controls (ratio 0.620, p = 0.001). (TIF) [file pone.0324143.s008.tif]
